# Differential Effects of Viruses on the Growth Efficiency of Freshwater Bacterioplankton in Eutrophic Relative to Non-Eutrophic Lakes

**DOI:** 10.3390/microorganisms11020384

**Published:** 2023-02-02

**Authors:** Angia Sriram Pradeep Ram, Télesphore Sime-Ngando

**Affiliations:** Laboratoire Microorganismes, Génome et Environnement, UMR CNRS 6023, Université Clermont-Auvergne, CEDEX, 63178 Aubière, France

**Keywords:** bacteria, viruses, viral lysis, bacterial growth efficiency, trophic status, French Massif Central lakes, microbial ecology

## Abstract

In aquatic environments, the consensus of viral impact on bacterial carbon metabolism with the nutrient environment as an important axis is limited. Henceforth, we explored the viral regulation of carbon-based bacterial growth efficiency (BGE) in a set of freshwater systems from French Massif Central, which were broadly classified based on two trophic statuses: eutrophic and non-eutrophic lakes. Comparative analysis showed that microbial abundances (viruses and bacteria) were 3-fold higher in eutrophic compared with non-eutrophic lakes, and so were bacterial production and viral lytic infection. The observed variability in BGE (10–60%) was explained by the uncoupling between bacterial respiration and production. Viruses through selective lysis of susceptible host communities had an antagonistic impact on BGE in the eutrophic lakes, whereas the release of substrates via viral shunt exerted a synergistic influence on the carbon metabolism of non-targeted host populations in non-eutrophic lakes. The decisive effect of the two individual processes (i.e., lysis and substrate release) on BGE was supported by regressions of bacterial abundance as a function of bacterial production, which is considered as a proxy of top-down processes. The role of viruses through their negative impact via mortality and positive impact via substrate supply can eventually have implications on carbon transfer through bacterioplankton in freshwaters.

## 1. Introduction

Heterotrophic bacteria, which represent the most important and active group among microbial communities, account for a significant fraction of plankton biomass in aquatic systems [1,2]. These prime biological components play a pivotal role in the biogeochemical cycles of organic matter and nutrients, essential to the ecological functioning of freshwater systems [3]. Our understanding of how these phylogenetically and metabolically diverse communities process organic matter inputs in lakes is fundamental in predicting the flow of energy through the aquatic food web [4,5]. The multiple roles of bacteria to convert the organic matter into their biomass (bacterial production, BP) and into nutrients (bacterial respiration, BR) which is controlled by bacterial growth efficiency (BGE), is imperative to comprehend their role in the carbon cycling [6]. Assessment of BGE is central to our understanding of microbial metabolism to evaluate the flow of organic carbon to higher trophic levels (biomass) and its energetic loss from the system (CO2).

Among the interacting forces explaining the variability in BGE, most attention has been given to the resource (bottom-up) availability [7,8,9], with a lesser focus on the prey–predator system. In recent years, aquatic viruses have been recognized as important and potential top-down agents which play vital roles in regulating carbon and nutrient fluxes, food-web dynamics, and microbial diversity [10,11]. Viral infection is known to vary systematically together with their principal hosts, bacteria along the lake trophic gradient, with reports of higher lysis in eutrophic compared with oligotrophic environments [12,13]. Viruses, through their host-specificity mechanism and density-dependent regulation, can transiently remove 20–60% of the daily bacterial population in lake ecosystems of varying trophic states [14]. Such a loss of bacterial cells that results in the alteration of bacterial diversity can have consequential effects on bacterial-mediated processes [15]. The variable impact of viruses on bacterial communities through their selective lysis (host specificity) and viral shunt (substrate supply) can have direct and indirect consequences on their structure and henceforth, on BGE. Although aquatic ecologists have underlined the importance of including viruses as one of the ecological determinants influencing BGE [16,17], fewer investigations have been done. Viral regulation of bacterial metabolism is less well-studied than resource availability. Studies conducted so far have revealed contrasting and complex scenarios concerning the viral impact on BGE in marine [18,19] and freshwaters [20,21].

Estimates of BGE that we measure are representative of the total bacterial population. Since it is now obvious that different members within bacterial communities are known to process organic matter at different efficiencies [6,22], selective lysis of susceptible hosts should impact the bulk BGE through its bacterial metabolic parameters. The present study is focused on a set of freshwater lakes from French Massif Central that are known to differ in terms of nutrient environment and henceforth provide a natural trophic gradient. Our previous studies from the lakes of the same geographical region have suggested lytic viruses exert a strong influence on bacterial diversity and richness [23]. However, it is not known whether the influence of viruses on BGE is the same for eutrophic and non-eutrophic systems.

Based on the published studies that viral lysis and BGE are known to differ with nutrient environments, we examined the relationship between them in lakes of French Massif Central, which were broadly grouped into eutrophic and non-eutrophic according to trophic state index (TSI) [24]. Investigations on the importance of nutrient environment as a major axis influencing the relationship between BGE and viral lysis is limited. Henceforth, we hypothesize that the variable viral lysis of bacterial community should invoke changes in BGE, and the association of viruses with BGE should differ between the lakes (eutrophic versus non-eutrophic). To test the hypothesis of viral influence on bacterial-mediated carbon fluxes, we collected data on bacterial metabolic parameters (BP and BR) together with viral processes related to their life strategies (lytic and lysogeny) in the pelagic realm of 25 temperate lakes of the contrasted trophy, distributed across a natural climatic gradient.

## 2. Materials and Methods

### 2.1. Study Sites and Sample Collection

Twenty-five lakes (natural and man-made) from French Massif Central that differ in hydrological and morphological characteristics were included in the sampling program (see Keshri et al. [23] for sampling location, detailed site description, and characteristics). Sub-surface lake water samples (0.5 m depth) were collected (in replicates that included three sampling operations) at their deepest point during the summer of 2012 (between 14th and 18th August) using a horizontal Van Dorn bottle (5L capacity). The collected samples were immediately pre-filtered through a 150 µm nylon mesh to eliminate larger organisms and poured into clean polycarbonate bottles. The samples were transported to the laboratory under cold temperature conditions in refrigerated boxes and processed immediately for water chemistry and microbiological parameters upon arrival. On each date, sampling at different lakes was planned and organised based on their proximities.

### 2.2. Limnological Variables

Water temperature and dissolved oxygen were measured using a field thermometer and Winkler’s method, respectively. pH was determined electrometrically using a pH meter (pH 1000L, VWR, Lutterworth, UK). Total organic carbon (samples passed through 150 µm nylon mesh) and dissolved organic carbon (filtered through combusted GF/F filter) together with total nitrogen were determined using a Shimadzu TOC-VCNP analyser (Tokyo, Japan) [25]. Water colour was estimated by measuring absorbance at 440 nm on 0.45µ filtered water samples with a 10 cm quartz cell on a Cecil CE2021 UV-Visible spectrophotometer (Cambridge, England). Absorbance measurements which were baseline-corrected using ultrapure water, were calculated according to Hu et al. [26]. Chlorophyll concentration (Chl a), which represents the biomass of phytoplankton in lakes, was determined by filtering 300–500 mL of water samples through 47 mm diameter Whatman GF/F filters. Pigments were extracted from these filters with 10 mL of 90% acetone in the dark at 4 °C overnight. The samples were then centrifuged at 3000 rpm for 15 min and the optical density (OD) of the supernatant was measured using a spectrophotometer at 630 nm, 645 nm, 663 nm, and 750 nm. Chlorophyll concentrations were calculated according to the recommended method of APHA [27]. Lakes were classified into eutrophic and non-eutrophic by a widely used method, namely, Trophic State Index (TSI) formulated by Carlson [24], which is calculated using three parameters: Secchi disc transparency, Chlorophyll content and total phosphate. TSI of above 50 and less than 50 were designated as eutrophic and non-eutrophic lakes, respectively.

### 2.3. Flow Cytometry Analyses: Enumeration of Viral and Bacterial Abundances

Viral and bacterial abundances were enumerated using a FACS Calibur flow cytometer (Becton Dickinson, Franklin Lakes, NJ, USA) equipped with an air-cooled laser ion laser emitting at 488 nm (power at 15 mW) with the standard filter set-up [28]. 2 mL aliquots of lake water samples were fixed with paraformaldehyde (final concentration 0.5%) for 30 min, then diluted with TE buffer (pH: 8.7) and stained with SYBR Green (final concentration of 1 × 10^−4^ of commercial stock, Molecular Probes, Eugene, OR, USA). The staining of water samples was carried out in the dark at 80 °C for 10 min for viral samples and at room temperature for 15 min for bacterial samples. Viral samples were allowed to cool at room temperature prior to flow cytometry analysis. The output data were analysed using Cell Quest Pro software (BD Sciences, San Jose, CA, USA, version 4.0). Viral and bacterial abundances were expressed as viral-like particles (VLP) mL^−1^ and cells mL^−1^, respectively.

### 2.4. Bacterial Production (BP), Respiration (BR) and Growth Efficiency (BGE)

For estimation of bacterial metabolic parameters (BP and BR), water samples were filtered through 1 µm polycarbonate filters (47 mm, Whatman, Maidstone, UK) either under gravity or using a hand-operated vacuum pump to minimise cell disruption. The chosen filters were efficient, which allowed more than 85% of the bacteria to pass through. Depending on the lake water, filters were often replaced to avoid clogging. All filtration equipment and silicon tubing were acid-washed prior to filtration to avoid contamination.

The modified dilution approach was applied to estimate bacterial production from bacterial-specific growth rate (µ) [29]. For each lake, 1 part of the water sample was diluted with 4 parts of 0.02 µm ultrafiltrate and dark incubated for a 24-h period at in situ temperature conditions. Specific growth rate (µ d^−1^) was calculated from the increase in the number of cells produced (Nt) during the incubation time (t), and cell concentration at the beginning (N0) as

µ = ln (Nt/N0)/t.

BP (cells L^−1^ h^−1^) was calculated as a product of µ and initial bacterial abundance and converted to C equivalents using a conversion factor of 20 fg per cell.

BR was determined in the lake water from a decrease in dissolved oxygen concentration over the course of 24-h incubation in dark using Winkler’s method [30]. Water samples were homogenously siphoned gently into 8 BOD bottles (150 mL capacity) without introducing air bubbles. Out of which, 4 bottles were immediately fixed with Winkler’s reagents (initial), whereas the remaining were dark incubated in temperature circulated water bath for a 24-h period (final) before fixation. BR was calculated from the differences in oxygen concentration before the initial and final based on end-point titration. Oxygen units were converted into carbon units by multiplying by the values with 0.375 and an RQ of 1.

We coupled estimates of BP and BR to calculate BGE using the equation (BP/BP + BR) × 100 [6].

### 2.5. Viral Lytic Infection

A transmission electron microscope (TEM) was used to estimate the frequency of visibly infected cells (FVIC) in order to calculate viral mediated bacterial mortality (VIBM) and also determine burst size estimates (BS) by counting the number of fully matured phage particles visible inside an infected bacterium [12]. Bacterial cells in glutaraldehyde (final concentration 1%) fixed samples were directly collected onto carbon-coated 400-mesh formvar TEM grids using a Beckman Coulter centrifuge equipped with a swing-out-rotor (SW 40 Ti) for 20 min at 70,000× *g*. The grids were stained with 2% uranyl acetate for 30 s, followed by three rinses with nanopure water. Excess liquid was immediately wicked away from the grids with absorbent paper and stored in a desiccator at room temperature until analyses. For each grid, a minimum of 400–600 cells were examined at 8000–40,000× magnification using a JEOL 1200 Ex TEM (JEOL Ltd., Tokyo, Japan) operated at an accelerating voltage of 80 kV. A bacterial cell was considered infected when at least 3 or more phages were visualised inside the cell. FVIC counts were converted to the frequency of infected cells (FICs) and viral-induced bacterial mortality (VIBM) using the empirical equations of Weinbauer et al. [31] (FIC = 9.524 × FVIC − 3.256) and Binder [32] (VIBM = (FIC + 0.6 × FIC2)/1 − 1.2 × FIC), which relates to the bacteriophage infection cycle.

### 2.6. Induction of Lysogens

The percentage lysogeny (FLC) was determined from the increase in viral abundance and decrease in bacterial abundance with the addition of an inducing agent Mitomycin C (1 µg mL^−1^, Sigma, MO, USA) in 20 mL lake water samples (in triplicates) incubated at in situ temperature conditions over 24-h period relative to controls without addition [33]. FLC was estimated as

FLC (%): 100[VAMC − VAC)/(BSMax × BAt0)].

where VAMC and VAC are the viral abundances in the mitomycin C treatment and control assays after incubation, respectively, BSMax and BAt0 correspond to maximum burst size (bacterial filled with viruses) and bacterial abundance at the start of the experiment. The average BS, which was determined for each lake by TEM analyses, was used for the calculation of per cent lysogeny. BA and VA were determined by flow cytometry analysis as previously described (See Section 4.2).

### 2.7. Data Analyses

Potential relationships between microbial and environmental data sets were tested by linear pairwise Pearson raw correlation analysis and stepwise multiple regressions. Data were log-transformed to stabilise the variance and attain normality. All statistical analysis was performed using Minitab Version 17 (Minitab Inc., State College, PA, USA). The level of significance considered was at α = 0.05.

## 3. Result

### 3.1. In Situ Environmental Parameters or Limnological Characteristics

The data on limnological characteristics (mean ± SD) of the investigated lakes were sorted into two groups, namely eutrophic and non-eutrophic, which are presented in Table 1. During the studied summer season, surface water temperature in the pelagic realm that varied between 17.5 °C and 25.3 °C was in accordance with the lakes distributed along a natural altitudinal gradient (352–1192 m above sea level). The mean lake water temperature that decreased with altitude had a close and strong relationships with the estimated air temperature (r^2^ = 0.71, *p* < 0.001). Oxygenic conditions prevailed among the lakes (>8.5 mg L^−1^), with pH ranging from neutral (7.3) to alkaline (9.8) conditions. The contribution of dissolved organic carbon (DOC = 2.5–13.6 mg L^−1^) from 67 to 90% of the total fraction (TOC = 3.7–15.1 mg L^−1^) could be linked to the variability of phytoplankton biomass (and correspondingly high particulate carbon) at different nutrient conditions. Total nitrogen varied by an order of magnitude among the lakes (range = 0.1–1.1 mg L^−1^). The measurement of water colour as a proxy of coloured dissolved organic matter (CDOM), which arrives due to microbial degradation of organic matter and possible input from terrestrial sources, was maximum in Lake Roziers (8.3 m^−1^), coinciding with a high concentration of organic carbon. Irrespective of lake trophy, CDOM was significantly correlated (*p* < 0.01) with DOC concentration. Chlorophyll concentration, an indicator of phytoplankton biomass, showed a large variability (coefficient of variation, CV = 81%) that was estimated between 1.2 and 48.5 µg L^−1^ in the studied lakes, indicating differing degrees of trophy. The highest chlorophyll concentration that was recorded in Lake Philippe did not yield the highest CDOM. Overall, chlorophyll was correlated (*p* < 0.01) with DOC. Among the environmental parameters, organic carbon and chlorophyll concentration showed significantly (*p* < 0.001) high values in the eutrophic compared with a non-eutrophic set of lakes.

### 3.2. Flow Cytometry Counts

**Figure 1:**
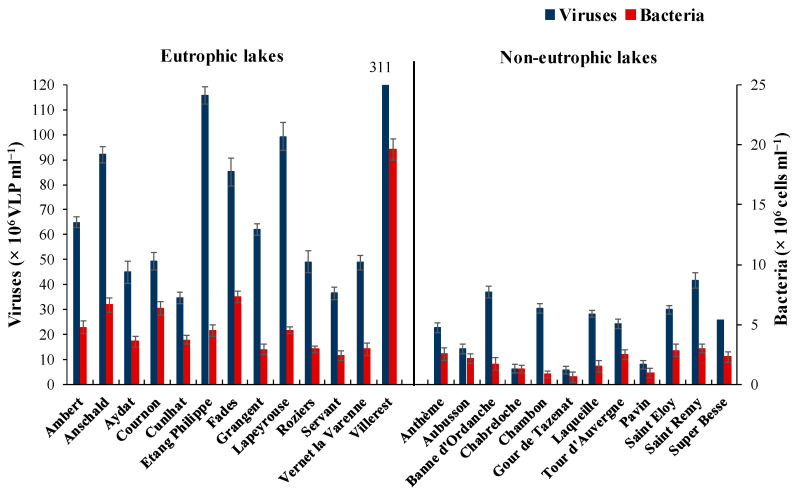
Flow cytometry abundances of viruses and bacteria in the eutrophic and non-eutrophic set of lakes. Data represents mean ± SD (n = 3).

Overall, flow cytometry analyses revealed viral (VA: 5.9 − 311.0 × 10^6^ VLP mL^−1^) and bacterial abundance (BA: 0.6 − 19.6 × 10^6^ cells mL^−1^) to vary by an order of magnitude (Figure 1), with their maxima observed in the same lake (Villerest). The mean abundance of VA and BA was 3.7 and 3-fold higher (*p* < 0.001) in the eutrophic lakes (VA: 84.1 ± 72.8 × 10^6^ VLP mL^−1^; BA: 5.6 ± 4.5 × 10^6^ cells mL^−1^) compared with non-eutrophic lakes (VA: 22.9 ± 12.0 × 10^6^ VLP mL^−1^; BA: 1.9 ± 0.8 × 10^6^ cells mL^−1^). Although the variability in VA and BA was large (CV > 80%) in the eutrophic than non-eutrophic lakes (CV < 50%), no significant differences in virus to bacteria ratio (VBR = 4.9–35.6) was evident. Irrespective of trophic status, VA was linearly correlated with BA (logVA = log1.17×BA − 0.02, r^2^ = 0.82, *p* < 0.001, Supplementary Figure S1) indicating that virioplankton in the lakes is to large extent represented by bacterial viruses or bacteriophages.

### 3.3. Bacterial Growth Efficiency

**Figure 2:**
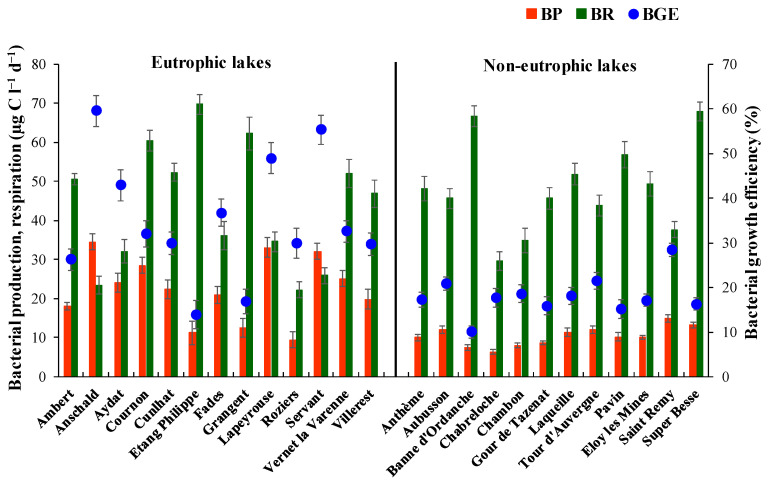
Estimates of bacterial production (BP), respiration (BR) and calculated growth efficiency (BGE) in the eutrophic and non-eutrophic set of lakes. Data represent mean ± SD (n = 3).

Estimates of bacterial growth efficiency (BGE) calculated from bacterial production (BP) and respiration (BR) measurements varied between 10% and 60% with eutrophic lakes (35.0 ± 13.6) recording 2-fold higher values on an average compared with non-eutrophic lakes (18.1 ± 4.4). The differences in BGE between the two group of lakes was attributed to fluctuations and differences in BP rather than BR. The mean value of BP in eutrophic and non-eutrophic lakes was 22.5 ± 15.7 and 10.4 ± 2.5 µg C L^−1^ d^−1^. The overall variation in BGE was largely explained by BP (y = 1.40x + 3.56, r^2^ = 0.84, *p* < 0.001). Unlike BP, which was dynamic, BR was more conservative (range = 22.2–69.8 µg C L^−1^ d^−1^) and did not vary significantly between the eutrophic and non-eutrophic lakes (Figure 2). On average, BR exceeds BP by 2.0 and 4.6-fold in the eutrophic and non-eutrophic lakes, respectively. The impact of prevailing lake environmental conditions on metabolic parameters, i.e., BP and BR, influenced the patterns of BGE in the grouped lakes. The differences in BGE were explained by a negative (*p* < 0.001) and insignificant (*p* > 0.05) relationship between BP and BR in the eutrophic and non-eutrophic lakes respectively.

### 3.4. Phage Life Strategy

**Figure 3:**
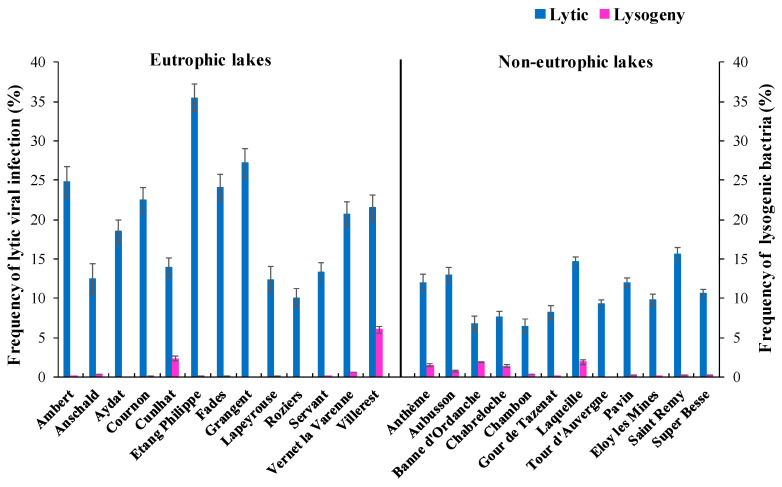
Frequency of lytic viral infection and lysogeny in the eutrophic and non-eutrophic set of lakes. Data represent mean ± SD (n = 3).

**Figure 4:**
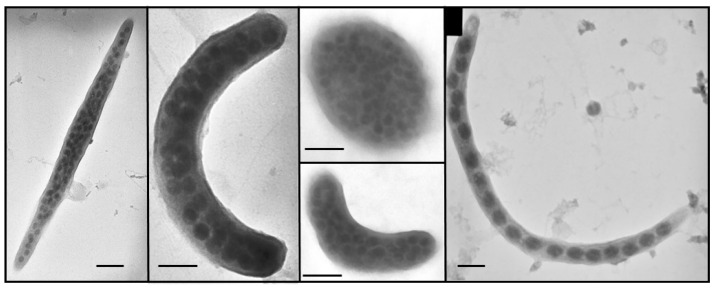
Electron micrographs of viral infected bacterial cells containing mature phage particles. Scale bar = 100 nm.

Lytic phage infection or active replication was more predominant than dormant lysogeny by two orders of magnitude among the lakes (Figure 3). The frequency of visibly infected cells, i.e., the proportion of bacteria containing mature phage particles which were visualized by TEM (Figure 4), varied from 1.0 to 4.1%. Based on this data, the percentage of infected bacterial cells by phages was calculated to range between 6.5% and 35.5% with a mean value of 19.8% and 10.5% that correspond to 30% and 12.7% of viral-mediated bacterial mortality in eutrophic and non-eutrophic systems respectively. The high viral lysis that was observed in Lake Philippe was found to coincide with the highest BR rate, which eventually yielded the lowest BGE among the investigated lakes. The number of visible intracellular phage particles or burst size estimates was significantly (*p* < 0.001) higher and variable in the eutrophic (range = 10 to ~350; mean BS = 36) compared with non-eutrophic lakes (range = 5–52; mean BS = 16). Per cent lysogeny determined from the increase in VA with mitomycin C treatment relative to uninduced control samples ranged from undetectable to 6% with no differences between the two groups of lakes. Although the values were low, per cent lysogeny was frequently detected in non-eutrophic (11 out of 12 lakes) compared with eutrophic lakes (4 out of 13). The highest per cent lysogeny values were recorded in eutrophic lakes with the highest microbial abundance.

### 3.5. Relationships between Variables

**Figure 5:**
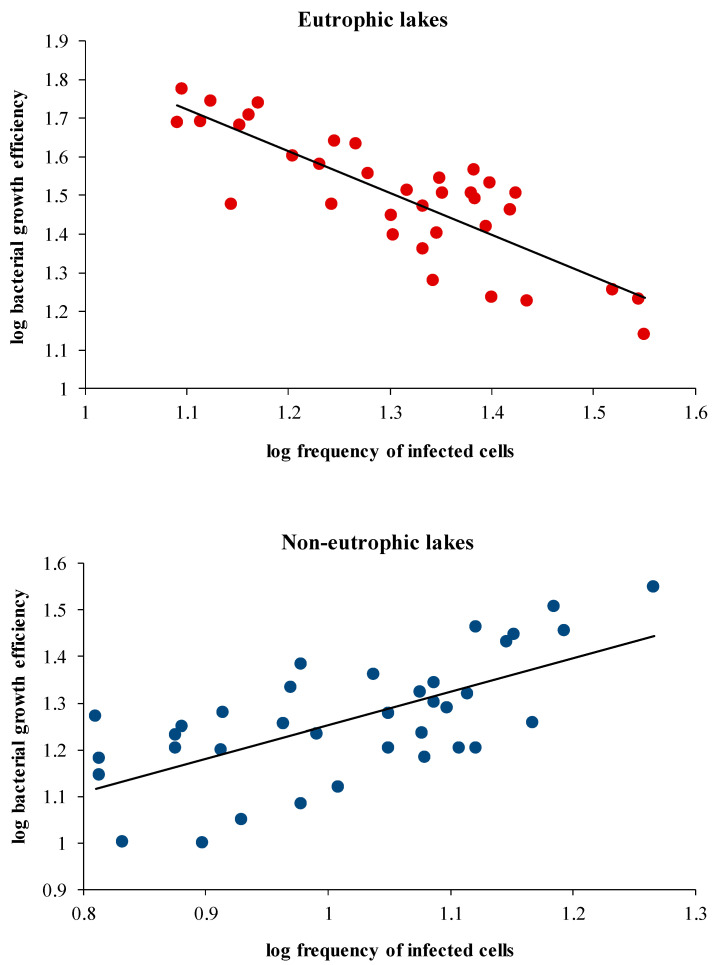
Regression analysis of frequency of viral infection (FIC) with estimates of bacterial growth efficiency (BGE) in the eutrophic [logBGE = −1.08 × log(FIC) + 2.92, r = −0.83, *p* < 0.0001] and non-eutrophic [logBGE = 0.72 × log(FIC) + 0.54, r = 0.66, *p* < 0.0001] set of lakes.

Correlation between variables exhibited different trends when the lakes were broadly grouped into eutrophic and non-eutrophic rather than analysing the whole data using an integrating approach (Table 2). Grouping generally led to a weakening of relationships between the variables, which could largely be attributed to the decrease in data points. However, some were not affected by it. In non-eutrophic lakes, a significant correlation (*p* < 0.05) of organic carbon and chlorophyll with BA and BP was observed. Regression analysis between BA and BP indirectly provides an indication of the factors (abiotic or biotic) potentially influencing the bacterial community. Accordingly, a positive correlation between BA and BP was observed in non-eutrophic lakes (*p* < 0.01), whereas no such trend was evident in the eutrophic lakes. Among the investigated lakes, lytic infection was never correlated with microbial abundances. Viral lysis was found to have a contrasting impact on BP, BR and BGE among the grouped lakes. However, such a relation was not evident when the whole data set was integrated together. Viral infection was positively and negatively correlated (*p* < 0.001) to BGE in non-eutrophic and eutrophic lakes, respectively (Figure 5), through its metabolic parameters, namely BP and BR. A significant correlation (*p* < 0.001) between lytic infection and BR was explained by a negative relationship between viral lysis and BGE in the eutrophic lakes.

## 4. Discussion

Differential effects of viral lysis on the carbon-based growth efficiency of natural bacterial communities (BGE) were evident in the French Massif Central lakes when they were broadly grouped according to the nutrient environment (eutrophy versus non-eutrophy). Viruses through selective lysis of susceptible host communities had an adverse impact on BGE in the eutrophic lakes, whereas the release of vital substrates via viral shunt exerted synergistic influence on non-infected populations in sustaining bacterial carbon metabolism. Contrasting scenarios of the two individual processes occurring in classified habitats were supported by regressions of bacterial abundance as a function of bacterial production, which is considered as a proxy of top-down processes [34,35]. The dual role of viruses as agents of mortality and/or stimulants of the growth of uninfected bacteria can have a variable influence on the bacterial-mediated carbon transfer to higher trophic levels in freshwater lentic systems.

### 4.1. Microbial Environment

Flow cytometry analysis which revealed 3-fold higher standing stock of viruses and bacteria in the eutrophic than non-eutrophic lakes, was largely explained by the differences in the dissolved organic carbon and chlorophyll concentration [13,36,37]. A strong positive correlation of viral abundance with bacteria rather than with chlorophyll concentration pointed out the dominance of bacteriophages (viruses which infect bacteria) in the viral population. Irrespective of lake trophy, a significant fraction (90%) of viral abundance was explained by bacteria alone, thus reflecting the trophic dependence of these microbes on one another [38,39].

The ability of viruses to face environmental challenges in aquatic habitats largely depends on their adopted life strategy, among which lytic infection was undoubtedly more predominant than lysogenic infection in the studied systems. Lytic infection, as evaluated from FVIC estimates (range: 1.0–4.1%) using the TEM approach, was comparable with literature reports from freshwater systems [40,41,42]. Our interpretation of viral-mediated bacterial mortality from FVIC by using the empirical models of Weinbauer et al. [31] and Binder [32], which essentially relates to the bacteriophage infection cycle, suggested that between 7% and 76% of bacterial production was destroyed among the investigated lakes [12,13,43]. Two-fold higher estimates of viral lysis in the eutrophic compared with non-eutrophic lakes are suggestive of their relative importance in influencing bacterial communities, which are known to increase with trophic status [23,44,45]. In eutrophic lakes, viral attacks on bacterial communities were more dependent on the density of susceptible bacterioplankton rather than the density of the whole host population, which was inferred from the lack of relationship between the percentage of infected cells and viral abundance. Percentage lysogeny was detected in more than half of the study sites irrespective of lake trophic status, with the highest value (6%) observed in the hypereutrophic Villerest reservoir, suggesting that certain bacterial groups tend to remain as prophage irrespective of lake trophy. Since freshwater bacterial communities are known to be nutrient limited at some part of the year, lysogens could temporarily be detected when the hosts are constrained by the availability of suitable substrates [12,46].

### 4.2. Variable Impact of Viral Lysis on BGE in the Classified Lakes

The transfer of organic carbon or energy to higher trophic levels in aquatic systems is ultimately dependent on the growth efficiency of the natural bacterioplankton. We carried out multiple correlation analyses to determine whether BGE was coupled to any of the physical, chemical, and biological variables available. Overall, the wide variability in the BGE (10–60%) in the studied systems was explained by the uncoupling between production and respiration, which confers advantageous to natural bacterioplankton in freshwater bodies [21,47,48] that are prone to fluctuating nutrient conditions caused by physical and climatic disturbances. Published reports on the factors influencing BGE in a particular system or set of systems have largely been attributed to their differences in nutrient concentration or availability [8]. Temperature, DOC, and chlorophyll concentration showed significant, positive covariation with bacterial parameters (abundance, production, and growth efficiency), suggesting bottom-up control of bacterial populations in the non-eutrophic lakes. However, high viral abundances accompanied by high viral infection together with a non-significant relationship of bacterial abundance with production and bottom-up forces (temperature, DOC, and chlorophyll) in eutrophic lakes lend support to a greater role and importance of viral lysis on BGE [18,20,21,49]: an issue that was previously raised in past studies [16,17]. We principally focused on lytic viruses, which through subsequent accelerated production of phage particles, directly contribute to viral shunt rather than dormant temperate viruses.

In agreement with our hypothesis, contrasting patterns of a significant negative and positive relationship between viral lysis and BGE were noticed when the lakes were grouped according to eutrophic and non-eutrophic status, respectively, instead of subjecting the whole data set to an integrated approach. Such an outcome can bring out the variable role of viruses in mediating microbial-driven processes such as carbon cycling in freshwater systems.

The putative adverse effect of viruses on BGE through their impact on bacterial metabolic processes (bacterial production and respiration) was targeted towards preferential lysis of specific host, which exhibited robust high growth rates [50,51]. Among the host community, the active high nucleic acid content bacteria, which are always more competitive in acquiring resources and have the high capacity to incorporate carbon, are vulnerable to viral infection [19,51]. Amidst high viral lysis, BGE was significantly high in the eutrophic lakes indicating that both control mechanisms (bottom-up and top-down) tend to operate simultaneously and work in concert in controlling bacterial community and function [52,53], which is supported by a narrow variation range in virus-to-bacteria ratio. Experimental and theoretical investigations from marine and freshwater systems have shown viruses to depress BGE at the community level either through selective lysis [51,54] or enhanced respiration rates in processing the lysis products [19,20,55]. Our previous studies in freshwater systems have indicated that a threshold value of 10% viral infection and a virus-to-bacteria ratio of >5 was sufficient to induce changes in bacterial community structure [23], which would eventually influence estimates of BGE.

In contrast to eutrophic, viral lysis yielded a significant positive relationship with BGE in the non-eutrophic lakes, which perhaps indicates that the nutrient spill as a consequence of host cell lysis could provide important substrates for the growth of certain bacterial groups during the period of nutrient limitation, similar to reports from freshwater systems [21,56]. In addition to viral lysis, protistan grazing activity can equally stimulate the growth of non-targeted bacterial communities through the supply of vital resources [57]. Experimental studies have indicated BGE to increase in marine microcosms supplemented with DOM originating from the bacterial community [58]. In a model system, viral lysis of a marine bacterium (*Cellulophaga* sp.) by a virus specific to bacteria has revealed viral lysates to be rich and contributed to a major fraction of dissolved free and combined amino acids (DFAA, DCAA) and D-amino acids which could be efficiently assimilated by the non-targeted to meet their nutrient demand [59], especially under nutrient-limited conditions [60]. FT-ICR MS analyses have shown viral-induced DOM to contain high molecular weight nitrogen compounds and when liberated into environment, it can be quickly utilized and transformed by aquatic bacteria, thus revealing its high labile nature [61,62]. Moreover, a theoretical study has indicated that BGE could increase if the viral lysis augments the ratio of exported carbon relative to the primary production-limiting nutrients, namely nitrogen and phosphorous [63]. In such a case, the virally infected cells form large particles that can be preferentially grazed by heterotrophic protists resulting in their higher growth [64] and increased transfer of energy to higher trophic levels.

The observation of positive (viral shunt) and negative (mortality) association of viruses on bacterial metabolism authenticate the importance of nutrient environment as an important axis. Pourtois et al. [65], through the multitrophic model, hypothesized that the influence of viruses on an ecosystem could vary depending on which nutrient is limiting (N or P). Different bacterial species are known to process organic matter at various efficiencies. Henceforth, suppression of a particular community could be beneficial to another; as a consequence, the positive and negative impact of viruses on BGE can vary among host populations. Therefore, viruses in either way (lysis or substrate supply) should impact BGE through changes in bacterial community structure, but the magnitude should largely depend on the prevailing nutrient conditions.

Viral infection is strongly dependent or regulated by host’s physiological state. However, its link with BGE is not clear owing to susceptibility or defensive characteristics towards infection. Experimental studies pertaining to the impact of viruses on BGE through concomitant changes in bacterial community structure with contrasting nutrient conditions are required to bring out the interplay between virus-bacteria interactions in freshwater systems with varying trophic status.

## Figures and Tables

**Table 1:** Limnological characteristics of the studied lakes grouped according to non-eutrophic (italics) and eutrophic (bold) status. Mean SD (n = 3).

Lake	Water Temperature (°C)	pH	CDOM (m^−1^)	TOC (mg L^−1^)	DOC (mg L^−1^)	TN (mg L^−1^)	Chlorophyll a (µg L^−1^)
*Antheme*	*20.4 ± 0.1*	*8.7 ± 0.1*	*1.8 ± 0.2*	*7.9 ± 0.8*	*5.2 ± 0.5*	*0.4 ± 0.04*	*13.6 ± 0.9*
*Aubusson*	*22.4 ± 0.2*	*7.7 ± 0.2*	*2.8 ± 0.3*	*8.2 ± 0.9*	*7.8 ± 0.6*	*0.7 ± 0.05*	*6.3 ± 0.8*
*Banne d’ordanche*	*19.3 ± 0.2*	*8.2 ± 0.1*	*0.7 ± 0.1*	*5.6 ± 0.4*	*3.8 ± 0.2*	*0.4 ± 0.02*	*5.6 ± 0.5*
*Chabreloche*	*20.5 ± 0.1*	*7.3 ± 0.2*	*2.8 ± 0.2*	*5.8 ± 0.5*	*5.3 ± 0.4*	*0.5 ± 0.05*	*3.7 ± 0.2*
*Chambon*	*21.3 ± 0.2*	*7.6 ± 0.2*	*0.9 ± 0.1*	*8.2 ± 0.9*	*4.7 ± 0.3*	*0.2 ± 0.02*	*4.1 ± 0.4*
*Gour de Tazenat*	*24.5 ± 0.3*	*8.5 ± 0.2*	*0.5 ± 0.06*	*6.4 ± 0.5*	*5.3 ± 0.4*	*0.3 ± 0.03*	*2.0 ± 0.2*
*Laqueille*	*17.5 ± 0.2*	*7.7 ± 0.3*	*0.6 ± 0.05*	*4.3 ± 0.3*	*3.2 ± 0.3*	*1.1 ± 0.09*	*10.9 ± 0.8*
*Tour d’Auvergne*	*21.6 ± 0.1*	*7.9 ± 0.1*	*0.5 ± 0.03*	*7.6 ± 0.5*	*5.4 ± 0.5*	*0.2 ± 0.02*	*13.1 ± 0.9*
*Pavin*	*20.9 ± 0.2*	*7.4 ± 0.1*	*0.5 ± 0.03*	*3.7 ± 0.2*	*2.5 ± 0.2*	*0.1 ± 0.01*	*1.2 ± 0.2*
*St.Eloy les Mines*	*24.5 ± 0.1*	*8.2 ± 0.1*	*1.2 ± 0.1*	*8.6 ± 0.8*	*7.4 ± 0.5*	*0.3 ± 0.02*	*3.8 ± 0.2*
*St. Remy*	*23.2 ± 0.2*	*8.2 ± 0.2*	*1.6 ± 0.2*	*8.4 ± 0.9*	*6.3 ± 0.4*	*0.7 ± 0.03*	*9.4 ± 0.8*
*Super Besse*	*18.8 ± 0.2*	*7.9 ± 0.2*	*0.7 ± 0.07*	*4.5 ± 0.3*	*3.5 ± 0.2*	*0.1 ± 0.03*	*9.2 ± 0.7*
**Ambert**	**23.9 ± 0.2**	**7.7 ± 0.1**	**1.2 ± 0.2**	**14.3 ± 1.1**	**10.3 ± 0.7**	**0.4 ± 0.02**	**8.8 ± 0.7**
**Anschald**	**21.9 ± 0.2**	**7.2 ± 0.2**	**1.2 ± 0.1**	**8.1 ± 0.7**	**8.5 ± 0.8**	**0.4 ± 0.02**	**11.2 ± 0.9**
**Aydat**	**23.1 ± 0.3**	**9.1 ± 0.2**	**0.7 ± 0.08**	**9.8 ± 0.9**	**6.6 ± 0.4**	**0.5 ± 0.02**	**15.6 ± 1.0**
**Cournon**	**24.2 ± 0.1**	**8.9 ± 0.3**	**0.5 ± 0.05**	**11.7 ± 0.9**	**7.7 ± 0.5**	**0.3 ± 0.01**	**9.5 ± 0.7**
**Cunlhat**	**21.6 ± 0.2**	**8.1 ± 0.2**	**3.0 ± 0.2**	**14.6 ± 0.9**	**9.2 ± 0.5**	**0.5 ± 0.02**	**19.9 ± 1.1**
**Etang Philippe**	**23.3 ± 0.1**	**7.5 ± 0.1**	**0.7 ± 0.05**	**13.7 ± 0.8**	**10.6 ± 0.5**	**0.5 ± 0.01**	**48.5 ± 1.5**
**Fades**	**23.4 ± 0.3**	**9.7 ± 0.2**	**2.3 ± 0.08**	**11.2 ± 0.8**	**8.5 ± 0.7**	**0.6 ± 0.02**	**17.0 ± 1.2**
**Grangent**	**22.3 ± 0.2**	**7.4 ± 0.2**	**1.8 ± 0.1**	**7.4 ± 0.5**	**5.8 ± 0.5**	**1.0 ± 0.08**	**11.6 ± 0.9**
**Lepeyrouse**	**24.4 ± 0.2**	**8.2 ± 0.1**	**0.7 ± 0.05**	**13.0 ± 0.8**	**11.2 ± 0.9**	**0.7 ± 0.05**	**21.4 ± 1.3**
**Servant**	**25.3 ± 0.3**	**7.6 ± 0.2**	**1.2 ± 0.02**	**12.1 ± 0.9**	**10.4 ± 0.7**	**0.6 ± 0.03**	**7.2 ± 0.5**
**Vernet la Varenne**	**22.8 ± 0.1**	**8.1 ± 0.2**	**1.4 ± 0.2**	**13.6 ± 0.7**	**9.7 ± 0.6**	**0.4 ± 0.02**	**13.3 ± 0.9**
**Villerest**	**24.8 ± 0.2**	**9.8 ± 0.2**	**1.6 ± 0.2**	**10.2 ± 0.8**	**8.6 ± 0.4**	**0.5 ± 0.02**	**25.8 ± 1.1**
**Roziers**	**21.8 0.3**	**7.1 ± 0.1**	**8.3 ± 0.7**	**15.1 ± 0.9**	**13.6 ± 1.0**	**0.5 ± 0.01**	**15.5 ± 1.3**

CDOM: coloured dissolved organic matter, TOC: total organic carbon, DOC dissolved organic carbon, TN: total nitrogen.

**Table 2:** Correlation matrix (Pearson r) between the key environmental (abiotic and biotic) variables. Values in bold, italics and in normal text represent eutrophic (n = 13), non-eutrophic (n = 12) and total lakes (n = 25), respectively.

Parameters	Temp	CDOM	DOC	Chl	VA	BA	FIC	BR	BP
**Temp**									
**CDOM**	NS								
**DOC**	**NS**/*NS* 0.61 ***	**NS**/0.88 *** 0.51 ***							
**Chl**	NS	NS	**NS**/*NS* 0.51 ***						
**VA**	**NS**/*NS* 0.38 *	NS	**NS**/**NS** 0.38 *	**NS**/*NS* 0.49 ***					
**BA**	**NS**/*NS* 0.41 *	NS	**NS**/0.53 * NS	**NS**/0.53 * 0.51 ***	0.94 ***/0.56 * 0.94 ***				
**FIC**	**NS**/*NS* 0.37 *	NS	**NS**/*NS* 0.37 *	**NS**/*0.61* 0.47 **	**NS**/*NS* 0.45 **	**NS**/0.58 * 0.42 *			
**BR**	NS	**NS**/−0.60 * −0.38 *	**NS**/−0.68 ** NS	NS	NS	NS	0.77 ***/*NS* NS		
**BP**	**NS**/*NS* 0.48 **	NS	**NS**/*NS* 0.51 ***	**NS**/0.68 ** 0.56 ***	NS	**NS**/0.69 *** 0.38 *	−0.87 ***/0.87 *** NS	−0.75 ***/*NS* NS	
**BGE**	**NS**/*NS* 0.47 **	NS	**NS**/0.61 * 0.56 ***	**NS**/0.60 * 0.50 ***	NS	**NS**/0.70 *** NS	−0.85 ***/0.66 ** NS	−0.94 ***/−0.72 *** −0.66 ***	0.92 ***/0.69 ** 0.91 ***

Temp: water temperature, CDOM: colored dissolved organic matter, DOC: dissolved organic carbon, Chl: chlorophyll a concentration, VA: viral abundance, BA: bacterial abundance, FIC: frequency of infected cells, BR: bacterial respiration, BP: bacterial production, BGE: bacterial growth efficiency. Level of significance: * *p* < 0.05, ** *p* < 0.01, *** *p* < 0.001. NS = values not significant.

## Data Availability

Data is contained within the article or Supplementary Materials.

## Supplementary Materials

The following supporting information can be downloaded at: https://www.mdpi.com/article/10.3390/microorganisms11020384/s1, Figure S1: Relationship between bacterial and viral abundance in the lakes of French Massif Central.
