# Supplementary material for: Differential Effects of Viruses on the Growth Efficiency of Freshwater Bacterioplankton in Eutrophic Relative to Non-Eutrophic Lakes
Source: Microorganisms. 2023 Feb 2;11(2):384. doi: 10.3390/microorganisms11020384 (PMC9966266; doi:10.3390/microorganisms11020384)

Supplementary Figure S1. Relationship between bacterial and viral abundance in the lakes of French Massif Central.

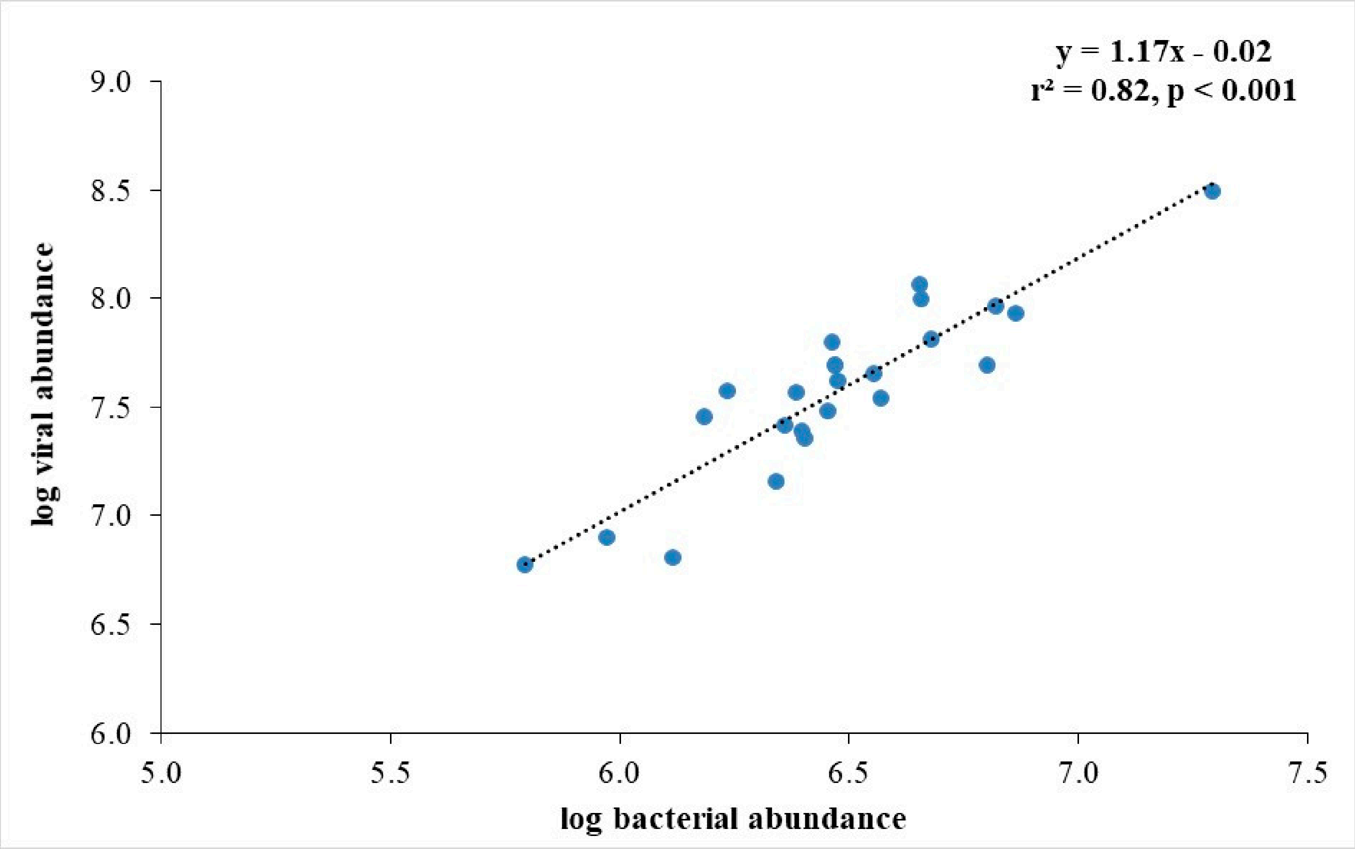

Supplement: Supplementary file 1 [file microorganisms-11-00384-s001.zip › microorganisms-2120368-supplementary.pdf]
